# A Newly Assigned Role of CTCF in Cellular Response to Broken DNAs

**DOI:** 10.3390/biom11030363

**Published:** 2021-02-27

**Authors:** Mi Ae Kang, Jong-Soo Lee

**Affiliations:** Department of Life Sciences, Ajou University, Suwon 16499, Korea; makang@ajou.ac.kr

**Keywords:** CTCF, DNA damage repair, homologous recombination

## Abstract

Best known as a transcriptional factor, CCCTC-binding factor (CTCF) is a highly conserved multifunctional DNA-binding protein with 11 zinc fingers. It functions in diverse genomic processes, including transcriptional activation/repression, insulation, genome imprinting and three-dimensional genome organization. A big surprise has recently emerged with the identification of CTCF engaging in the repair of DNA double-strand breaks (DSBs) and in the maintenance of genome fidelity. This discovery now adds a new dimension to the multifaceted attributes of this protein. CTCF facilitates the most accurate DSB repair via homologous recombination (HR) that occurs through an elaborate pathway, which entails a chain of timely assembly/disassembly of various HR-repair complexes and chromatin modifications and coordinates multistep HR processes to faithfully restore the original DNA sequences of broken DNA sites. Understanding the functional crosstalks between CTCF and other HR factors will illuminate the molecular basis of various human diseases that range from developmental disorders to cancer and arise from impaired repair. Such knowledge will also help understand the molecular mechanisms underlying the diverse functions of CTCF in genome biology. In this review, we discuss the recent advances regarding this newly assigned versatile role of CTCF and the mechanism whereby CTCF functions in DSB repair.

## 1. Introduction

Our genome is continually assaulted by environmental genotoxic agents, such as ultraviolet (UV) light, ionizing radiation (IR), and numerous chemicals [1,2,3,4], and also by various endogenous factors, such as free radicals and reactive species, resulting from the cellular metabolism [5,6,7]. Furthermore, genome instability can arise from replication-fork stalling and collapse, driven by improper DNA secondary structures and by transcription-associated R-loops, which often form during DNA replication. Un- or mis-repaired DNA lesions cause mutations or chromosomal rearrangements, which can lead to genetic disorders or diseases [5].

**Figure 1:**
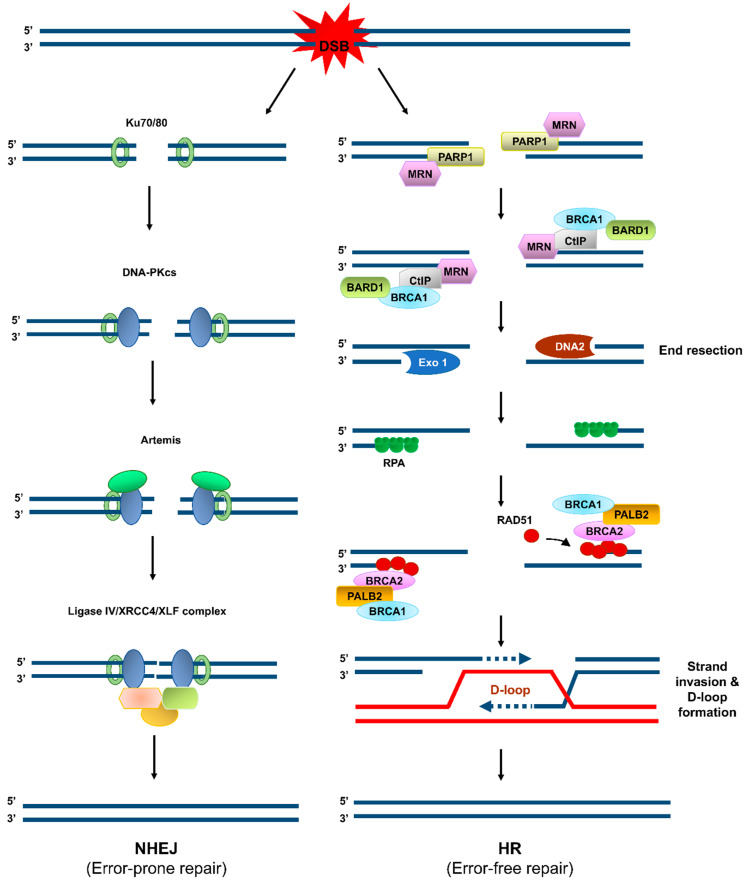
Error-prone and error-free pathways of DNA double-strand break (DSB) repair. When a DSB is formed, it is repaired through one of two major repair mechanisms: non-homologous end joining (NHEJ, left) and homologous recombination (HR, right). In the NHEJ pathway, the DNA ends associate with the Ku70/80 heterodimer and DNA-dependent protein kinase catalytic subunit (DNA-PKcs), which further recruits Artemis, XRCC4, and Ligase IV to trim the DNA ends nucleolytically, fill in the DNA gap formed, and then ligate the two ends. NHEJ is a highly efficient DSB repair pathway, but it occasionally entails deletion or insertion of several nucleotides. In the HR pathway, PARP1 binds to the DSBs, and the Mre11/Rad50/Nbs1 complex is recruited to the DSBs, followed by recruitment of CtIP and BRCA1/BARD1 to initiate DSB end resection. Extensive end resection is catalyzed by EXO1 and DNA2/BLM to yield long single-stranded DNA (ssDNA) tails, which become coated by replication protein A (RPA). The BRCA2/PALB2/BRCA1 complex facilitates replacement of RPA with RAD51. The RAD51–ssDNA filament seeks the homologous DNA sequence on its sister chromatid or on the homologous chromosome and catalyzes the formation of displacement loop (D-loop). DNA polymerization occurs by using the sister chromatid as a template, and the resolution of the resulting complex produces an accurate copy of the template.

Among the vast number of constantly occurring DNA lesions, DNA double-strand breaks (DSBs), indirectly or directly generated by IR, UV, or chemical agents, are the most harmful ones and the common causative factors for genome instability [5]. DSBs are primarily repaired through two major pathways: non-homologous end joining (NHEJ) and homologous recombination (HR) (Figure 1) [5,8]. NHEJ is an error-prone repair pathway, because its DNA products mostly harbor deletion/insertion mutations resulting from the trimming of the ends of the DSBs and the subsequent gap-filling and ligation (Figure 1). NHEJ occurs independently of a homologous template. It is active throughout the interphase (G_1_, S, and G_2_ phases), especially during G_1_. By contrast, HR requires the homologous sister chromatid and thus occurs during S and G_2_ phases of the cell cycle [8]. HR is an error-free DSB repair pathway, because it relies on the homology between two DNA strands [8]. However, HR is not always error-free and NHEJ is not always error-prone. Genome instability (bad outcomes) can arise from abortive HR intermediates during DSB repair or replication restart [9] and the end-joining accuracy (good outcomes) can arise from the DNA end structures that permit direct ligation of compatible DNA ends [10]. The choice of repair pathway for a DSB between HR and NHEJ specifies the repair outcome regarding genome integrity. To elude DSB-induced cytotoxicity and guard the genome, the HR-mediated DSB repair pathway is ideal, because HR can restore the DNA sequence before any DNA damage occurs and therefore ensures the correct repair of a DSB.

Studies on DSB repair mechanisms and pathway choice between HR and other DSB repair pathways, including NHEJ, are noteworthy as they are relevant to the maintenance of genome integrity and to the biology of cancer, senescence, and aging. During HR-mediated DSB repair (Figure 1), DSBs are targeted by a complex array of proteins, including the MRE11–RAD50–NBS1 (MRN) complex, which further recruits numerous other proteins, such as CtIP, to conduct the nucleolytic degradation of the ends of the DSB, known as end resection. Importantly, this process passes the DSB to the HR repair pathway, as the initial step of HR. MRE11 endonuclease, in collaboration with phosphorylated CtIP, cleaves the 5′-terminated DNA strands near DSBs, followed by BLM/DNA2- and Exo1-catalyzed extensive end resection, to yield 3′ single-stranded DNA (ssDNA) tails [11]. The ssDNA tails are instantly loaded with replication protein A (RPA) [12] and then displaced with the recombinase RAD51 to form a helical nucleoprotein filament [13]. This filament seeks the homologous DNA in the sister chromatid or homologous chromosome. After identifying the homologous DNA, the filament invades it and thereby forms the heteroduplex displacement loop (D-loop). This process is followed by DNA synthesis within the D-loop and resolution of the intermediate DNA structures to complete the repair [14,15].

The roles of CCCTC-binding factor (CTCF) in gene expression and genome organization are well-characterized; however, in addition, several lines of evidence have recently indicated that CTCF is also involved in DSB repair as an HR protein [16,17,18]. This highly conserved protein was discovered as a transcriptional repressor of the chicken *c-myc* gene and named so because it binds to the regularly spaced CCCTC repeats in the *c-myc* promoter region through its 11 zinc-finger domains. Initially characterized as a dual-purpose transcriptional factor with both activator and repressor roles in gene expression, CTCF was subsequently found to engage in insulation to interfere with enhancer–promoter interactions or to define the boundaries between euchromatin and heterochromatin domains. Later, genome-wide CTCF-localization analyses, and approaches related to chromosome conformation capture (3C), helped understand how CTCF plays an exceptional role in three-dimensional genomic organization through its ability to establish long-range loops in collaboration with cohesin, in which CTCF acts as anchors for loops presented by cohesin-mediated loop extrusion (see the next section entitled “*A Chromatin Organizer for Global Genome Architecture*”). As this pleiotropic protein seems to be involved in more genomic events than what is known, its role in DNA repair extends beyond the role of an insulator protein or genomic architectural protein via long-range chromatin interactions. Here, we review what we have learned about CTCF so far, with a focus on its newly assigned role in DSB repair as a guardian of the genome.

## 2. The Multifunctional CTCF Protein and Its Classical Roles

**Figure 2:**
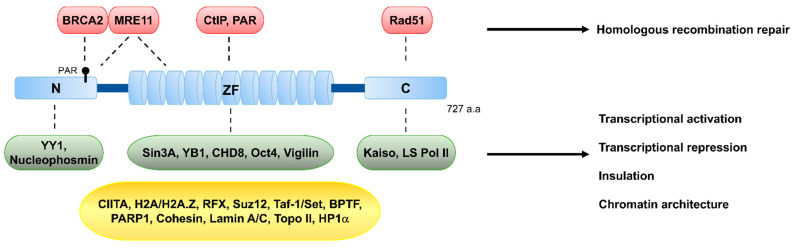
Schematic structure of CCCTC-binding factor (CTCF) and its known interacting partners. CTCF is composed of three structurally distinct regions, an N-terminal (N), 11-zinc-finger domain (ZF), and a C-terminal (C). CTCF harbors 11 repeats of zinc-finger motifs, which confer the ability to bind to the 11~15-bp consensus sequence and a vast array of variants of CTCF-binding sites, and also to interact with various proteins involved in DSB repair, such as MRE11 and CtIP, or with chromatin remodeling and transcriptional factors, such as Sin3A, CHD8, and Oct4. The N-terminal region interacts with BRCA2, which is a protein involved in DSB repair, and with the transcriptional factor YY1. The C-terminal region interacts with the recombinase RAD51. The CTCF-interacting proteins are listed in the colored boxes. The protein interactors in red (DSB repair factors) and green (transcription and insulation) boxes interact with CTCF through its N, ZF, or C regions. The protein interactors in the yellow box, which are involved in transcription, insulation, and chromatin organization, interact with CTCF, but the regions of CTCF responsible for the interactions are elusive. See Table 1 for the list of additional protein interactors.

The 82 kDa CTCF protein is highly conserved in most bilateral animals and is ubiquitously expressed in the nucleus across all tissue types. Between its N-terminal and C-terminal regions, CTCF contains 11 central zinc-fingers (Figure 2), through which it binds to DNA. Engagement of these zinc-fingers in various combinations enables CTCF to bind to a wide range of DNA sequences [19,20,21] (see later). Given its ability to bind to variant sequences via different zinc-finger combinations, various combinations of the 11 zinc-fingers may take part in versatile interactions with various proteins. Additionally, interaction of CTCF with variant DNA sequences diversely changes the conformation of CTCF, and these conformational changes likely influence its interaction with diverse nuclear proteins [22]. This mechanism, whereby the differentially combinatorial zinc-finger binding to diverse sequences may facilitate engagement with various binding partners [16,17,18,23,24,25,26,27,28], diverse post-translational modifications, and ultimately confer CTCF with multiple functional roles, emphasizes the versatile functions of CTCF. We will first briefly introduce the classical roles of CTCF, before reviewing the current information about its role in guarding genome integrity.

### 2.1. A Transcriptional Repressor or Activator

As briefly mentioned above, CTCF was first characterized as a transcriptional repressor of the *c-myc* gene [31,32] and subsequently found to be thyroid hormone-responsive [33,34]. Afterward, CTCF was also isolated as an activator of the amyloid β protein precursor gene [35].

### 2.2. An Enhancer Blocker or Helper

CTCF can serve as an enhancer blocker at two extensively characterized loci that were seminal for the characterization of the functions of CTCF for the *β-globin* and *H19-insulin-like growth factor 2* (*Igf2*) loci. A cis element of the *β-globin* locus, identified as an insulator responsible for blocking the enhancer activity in this locus, has been found to be a binding site of CTCF and requires CTCF-binding for its insulator activity [36]. The chromosome conformation capture (3C) and relevant analyses have revealed that long-range interactions between the elements of the *β-globin* gene and the locus control region (LCR) involve CTCF through chromatin looping to block the enhancer activity. Collectively, insulators are DNA elements that play a key role in preventing the inappropriate interactions between both adjacent and distant genomic regions [22,37,38]. The insulator function is mediated by regulatory proteins that bind to the insulator sequences. Whereas many insulator-binding proteins are characterized in *Saccharomyces* and *Drosophila*, CTCF is the only known insulator-binding protein known in vertebrates [39,40,41]. CTCF plays an enhancer-blocking role by preventing the communication between enhancers and irrelevant promoters [22,36,37,38,39].

In the *H19*-*Igf2* locus, the *H19* and *Igf2* genes are separated by the imprinting control region (ICR). This region is a binding site of CTCF and can be methylated. The DNA methylation status of the CTCF binding sequences within the ICR determines whether CTCF binds to the ICR because CTCF can only bind to the unmethylated ICR on the maternal chromosome, thereby blocking the interaction of the *H19*-proximal enhancer with the *Igf2* promoter and ultimately repressing the *Igf2* gene [42,43,44]. Conversely, CTCF does not bind to the methylated paternal ICR, and therefore the corresponding enhancer is not blocked by CTCF and the insulator ICR, consequently activating the transcription of *Ifg2*.

The role of CTCF as an enhancer-inducer was appreciated later than its role as an enhancer-blocker. Observations from studies using the chromosome conformation capture carbon copy (5C) technique or chromatin immunoprecipitation (ChIP)-sequencing analyses show that most long-range interactions between distal enhancers and promoters are not blocked by the presence of CTCF-binding sites and, instead, proficient enhancers are enriched for CTCF and active-chromatin histone modifications, such as H3K4 methylation (H3K4me1/2) and H3K27 acetylation (H3K27ac), suggesting that CTCF mostly facilitates the interactions between enhancer elements and promoters [45]. 

### 2.3. A Regulator of Transcriptional Pausing and Alternative Splicing

So far, we have taken a look at roles of CTCF in transcriptional regulation at the initiation step. Accumulating evidence suggest that CTCF regulates the transcriptional processes downstream of the initiation. Indeed, CTCF binds to the 5′-UTR and introns of genes including the *Myb* locus and it regulates polymerase II pausing and alternative mRNA splicing [46].

### 2.4. A Regulator of Somatic V(D)J Recombination at Antibody and Antigen Receptor Loci

The role of CTCF in short/long-range and intra/inter-chromosomal chromatin interactions may extend beyond its classical attributes including transcriptional regulation, genome imprinting, and genome architecture (see below), because CTCF is involved in V(D)J recombination during adaptive immune response. Immunoglobulin (Ig) and T cell receptor (TCR) loci are made up of multiple copies of variable (V), diversity (D), joining (J) and constant gene segments that encompass large genomic regions [47]. During the early stage of B and T cell maturation, V(D)J recombination begins with V, D, and J segment choice across multiple gene segments in a nearly random fashion and rearranges the segments in one unit, ultimately resulting in various amino acid sequences of Igs and TCRs that allow for antigen receptor diversity [48]. The process of V(D)J recombination is mediated by a diverse collection of enzymes that involves recombination activating genes 1/2 (RAG 1/2 endonucleases), terminal deoxynucleotidyl transferase (TdT), Artemis nuclease (one of the non-homologous end joining (NHEJ) pathway for DNA repair), and several NHEJ tools such as DNA-dependent protein kinase (DNA-PK) and ligase [49]. Like no other key V(D)J recombination proteins, CTCF may regulate lineage- and developmental stage-specific segment choice through its ability for chromatin interactions during V(D)J recombination. CTCT binding to CTCF-binding elements (CBE) in Ig locus facilitates V-D-J rearrangement by impeding cohesin-driven RAG chromatin scanning across the Ig locus via loop extrusion [50]. In progenitor B cells, *Igh* V(D)J recombination is initiated by RAG binding to a J_H_-recombination signal sequence (RSS) within a recombination center (RC), followed by RAG scanning for convergent D-RSSs across chromatin presented by cohesin-mediated loop extrusion and D-J_H_ joining. Then, further RAG scanning from the recombined DJ_H_-RC-RSS to convergent V_H_-RSSs is impeded by CTCF binding to D-proximal CBEs, which promotes robust V_H_-DJ_H_ recombination [51]. These recent studies suggest that V(D)J recombination is governed by the formation of long-range chromatin loops by CTCF and cohesin.

### 2.5. A Chromatin Organizer for Global Genome Architecture

Accumulating genome-wide studies and chromosome conformation capture-based analyses highlight evidence for CTCF-mediated intra- and inter-chromosomal interactions and support a primary role for CTCF in the global organization of chromatin architecture. Studies for mapping genome interactions, using Hi-C, one of the chromosome conformation capture technologies, unveil the 3-dimensional genome architecture of higher eukaryotes that dictates its function. 3D genome organization is governed by both the co-segregation of active and inactive chromatin with its histone markers into “compartment domains” (1~10 Mb) as well as the formation of long-range chromatin loops into “loop domain” (<1 Mb), that is, topologically associating domains (TADs).

TADs are defined by a high frequency of interactions between genomes within TADs and a low frequency of interactions between adjacent domains. TAD boundaries are gene-enriched regions that are proficient for transcriptionally active genes and highly populated with binding sites for chromatin architecture proteins, CTCF and rad21 homologue (RAD21, also known as SCC1), a cohesin subunit, that is also a double-strand break repair protein. Cohesin complex containing RAD21 and CTCF cooperate to organize the 3D architecture of the genome in a way that cohesin facilitates the folding of genome into loops that are anchored by CTCF [29,52]. Long-range chromatin loops are formed via both loop extrusion by cohesin and loop anchoring by CTCF, leading to the formation of TADs. Loop extrusion is mediated by cohesin, in which cohesin binds to chromatin, reels and extrudes chromatin as loop. CTCF bound to chromatin can act as impediments to cohesin-mediated loop extrusion and define loop anchors, thereby contributing to TAD bordering in conjunction with cohesin proteins. 

This model is supported by degradation or depletion of RAD21 and CTCF loss [30,53,54]. Degrading RAD21 results in loss of loops and depletion of CTCF markedly decreases CTCF-anchored loops. CTCF contributes to the cohesin positioning/localization at CTCF-binding sites (including TAD boundaries) and stabilize it on chromatin. In the next section entitled “Future Questions and Perspectives”, we discuss whether CTCF, through its ability to localize cohesin on chromatin and its function in 3D genome organization, helps channel DSBs into the HR repair pathway or, anything else, regulate DNA damage response and genome integrity. 

## 3. Updated Role of CTCF in Genome Integrity

### 3.1. How and Why Does CTCF Accumulate at DNA Llesions?

Evidence to date supports that CTCF is recruited at DSB sites upon DNA damaging, in addition to the approximately 55,000~65,000 sites in mammalian genome that CTCF recognizes and binds through its zinc fingers. It now appears that the DNA damage-responsive CTCF recruitment does not rely on the wide range of its original binding DNA module sequences, because CTCF is able to locate at random DSB sites across the genome induced by micro-irradiation as well as at specific but CTCF-binding site-independent DSBs induced by zinc-finger nucleases (e.g., FokI), homing endonucleases encoded either within introns or as self-splicing, similar to transposons (e.g., I-PpoI or I-SceI), and restriction enzymes such as AsiSI [16,17,18,55]. Like many other DSB accumulating proteins, CTCF recruitment depends on ATM kinase activity, suggesting that CTCF recruitment to DSB sites requires DNA damage response (DDR) signaling initiated by ATM [18]. CTCF is recruited at multiple DSB sites that are induced by AsiSI/I-PpoI or micro-irradiation and scatted throughout the genome, and one single DSB site within the genome of one cell induced by the DSB induction systems using I-SceI or FokI, of which the one enzyme recognition site must be artificially introduced into the genome. Together, these results are suggestive of the DSB recruitment of CTCF in a sequence-independent manner, in response to DNA damage.

Elegant studies, following the recruitment kinetics for CTCF upon DSB induction at micro-irradiated chromatin [16,17,18,55] have revealed that CTCF is rapidly recruited following laser micro-irradiation in a manner that depends on MRE11 or poly (ADP–ribosyl)ation modification. Knockdown of any protein of MRE11–RAD50–NBS1 (MRN) complex that binds eagerly and quickly to DSBs and plays a crucial role in DNA damage response markedly reduces CTCF recruitment at DSBs. In parallel, PARP1 inhibitors to suppress poly (ADP-ribose) (PAR) synthesis remarkably abolishes the DSB recruitment of CTCF. According to these data, it appears likely that CTCF is located at DNA lesions without its direct binding to broken DNA, because the CTCF enrichment at DSBs requires MRN complex and PAR. Because the micro-irradiation assay can clearly distinguish true CTCF proteins enriched at sites of DNA lesions, we could rule out the possibility that the high frequency of CTCF-binding sites covers up throughout the genome and CTCF is an abundant nuclear protein across many or all cell types, which may make it difficult to distinguish the CTCF proteins adjacent or close to sites of DNA (by chance) from the true CTCF proteins recruited at DNA lesion sites following laser micro-irradiation (in response to DNA damage).

Notably, CTCF recruitment to the micro-irradiated chromatin or other cases of DSBs is mediated through its zinc finger domain that is originally used for its direct binding to the classical CTCF consensus sequences. Several studies using truncation mutants of CTCF protein found that only the zinc finger domain, but not the N- or C-terminal regions, is able to locate at the sites of DNA damage, in a manner similar to that of the full length of CTCF, supporting that the zinc finger domain is responsible for the rapid recruitment of CTCF to sites of DNA lesions. In parallel, a couple of studies have reported that CTCF recruitment at the DNA lesion is mediated through interactions between CTCF and its partners (such as three proteins of MRN complex or PAR) through its zinc fingers. For example, knockdown of any of MRE11–RAD50–NBS1 or PARP1 inhibitor leads to loss of recruitment of the zinc finger domain, just like the full-length of CTCF. As discussed above, CTCF’s ability to display its vastly diverse functions has been likely attributed to combinatorial engagement of different zinc fingers for both its DNA binding and protein interaction (or PAR recognition and binding). This structural feature increases the likelihood that CTCF’s interaction with MRN complex or PAR via its zinc finger domain would promote access to CTCF proteins at the DSBs, which account for the indirect recruitment of CTCF at broken DNA to faithfully maintain genome integrity.

### 3.2. Colocalization of CTCF with γ-H2AX Chromatin to Delimit γ-H2AX Chromatin Domain

**Figure 3:**
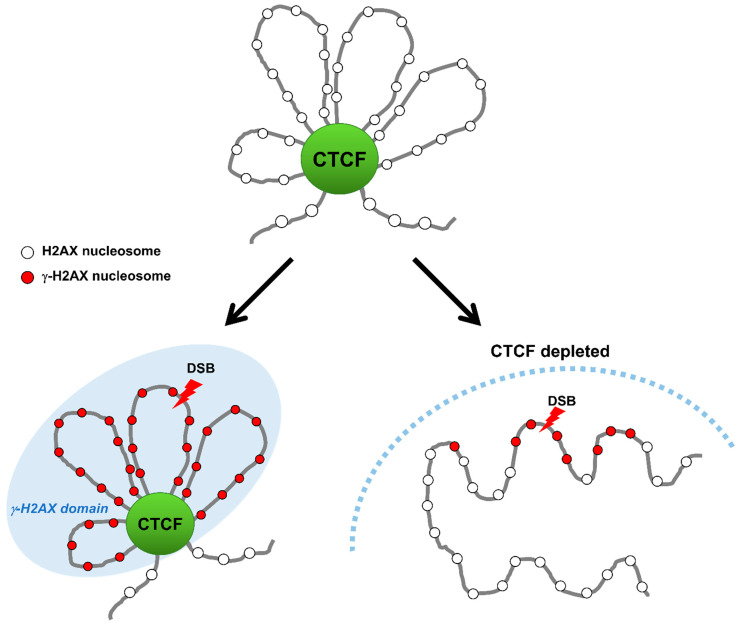
CTCF establishes γ-H2AX domains during DSB repair. Upon DNA damage, CTCF binds to DSBs and facilitates the formation of γ-H2AX domains by functioning as a domain-barrier protein. CTCF depletion destroys the higher-order chromatin structure and causes the γ-H2AX foci to spread and the DNA repair to be impaired.

**Figure 4:**
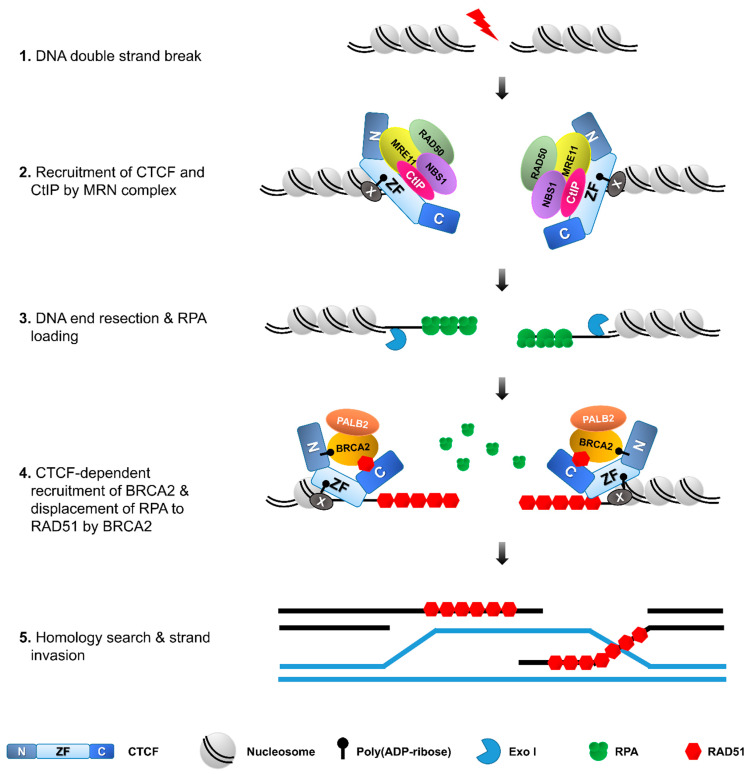
CTCF facilitates homologous recombination repair. During DSB repair, CTCF is recruited to DSBs in a PAR- or/and MRE11-dependent manner and, in turn, recruits CtIP. The MRE11–CtIP complex induces DNA end resection, followed by the loading of RPA onto the resultant single-strand DNA. CTCF also recruits BRCA2 to DSBs in a PARylation-dependent manner. CTCF and BRCA2, in turn, recruit RAD51, which forms filaments to allow strand invasion and homologous recombination to complete the DNA repair.

Furthermore, CTCF accumulated at DSBs is colocalized with the phosphorylated H2AX histone variant on serine 139 known as γ-H2AX on the delimited DSB chromatin, and many other DNA damage-responsive proteins (Figure 2, Figure 3 and Figure 4, Table 1). Remarkably, the accumulation of CTCF on the DSB chromatin after damage follows a near analogous pattern to the MRN complex that plays a critical role in the initial steps during the cellular DNA damage response prior to DNA repair by HR or NHEJ and facilitates initiation of DNA end resection to channel DSBs into HR [16] (Figure 4). Several studies provide strong evidence supporting that recruitment of CTCF at DNA lesions takes place within 30 s after DNA damage at an early stage of DDR [16,55].

By employing chromatin immunoprecipitation (ChIP) in combination with high-throughput sequencing (ChIP-seq), Natale et al. reported that, despite the conserved CTCF-binding sites across various cell type, γ-H2AX-rich chromatin domains are flanked by CTCF-binding sites before or during DNA repair, and both borders of a γ-H2AX chromatin domain are strongly enriched with CTCF-binding sites compared to the interior region of γ-H2AX chromatin domains [56]. In agreement with γ-H2AX distribution delimited by CTCF-binding sites, SMC3 was previously reported to antagonize H2AX spreading at cohesin-bound genes [57].

In agreement with their TAD bordering function in genome architecture, the enrichment of CTCF and cohesin at borders of both the TAD and γ-H2AX chromatin domains may have a causal role in defining their establishment [56,58]. A topological study for γ-H2AX landscape in which DSBs within one TAD confine γ-H2AX domains to inside of the TAD, and DSBs near a TAD border generates asymmetric γ-H2AX domains to preclude the γ-H2AX domains beyond the TAD borders, has further reinforced the importance of TAD borders formed by CTCF and cohesin for the γ-H2AX domain establishment [58,59]. This tempting hypothesis is that CTCF could serve as a boundary protein to confine γ-H2AX foci within damaged DNA regions by binding to boundaries of DNA lesions. Depletion of CTCF causes an increase in the size of the γ-H2AX chromatin region, implying that the γ-H2AX chromatin region can spread over outside the demarcated domain and expand the DDR region in the absence of CTCF [55]. This immediately raises three key questions: What it the function of CTCF-mediated demarcation of γ-H2AX chromatin domain? What enables CTCF binding to borders of γ-H2AX chromatin domain and how is this done? Anything else to engage in bordering for γ-H2AX chromatin domain as a border protein? However, this finding offers the possibility for an important mechanistic link between CTCF boundaries and DDR via protection/delimiting against the spread of the γ-H2AX chromatin domain. This result indicates that CTCF may accurately establish the spatial organization of the DDR by limiting the DDR area to γ-H2AX domains and facilitating access of DDR factors to the domain interior, although the accurate role of CTCF at borders of γ-H2AX chromatin domain and its underlying mechanism remain unclear.

### 3.3. A Promoter of DNA Repair via HR and Beyond

What is the role for CTCF at the interior and borders of the γ-H2AX chromatin domain (the so-called γ-H2AX foci)? Recently, several studies demonstrated a novel assignment of CTCF in HR through its ability to interact with crucial HR proteins including BRCA2, Rad51 and CtIP, and sequentially recruit them onto DSBs [16,17,18]. DNA repair by HR commences with the formation of extensive 3′-overhang single stranded DNA (ssDNA), which requires the recruitment of MRE11 and CtIP at the DSBs and activation of the endonuclease MRE11 in conjunction with its cofactor CtIP to create short 3’-ssDNA overhangs, and then involvement of a DNA exonuclease EXO1 and a helicase BLM/a DNA endonuclease DNA2 to generate long-range end resection [60,61]. Hwang et al. reported that exposure to etoposide or γ-irradiation (IR) promotes the interaction between CTCF and MRE11 via the N-terminal and zinc-finger domain of CTCF and N-terminal domain of MRE11 [16]. Because CTCF interact with MRE11 upon DNA damage and MRE11 binds avidly to double-strand breaks (DSB), CTCF is rapidly recruited to DSB sites in an MRE11-dependent manner. Depletion of MRE11 abolishes CTCF recruitment to the DNA damage sites, while depletion of CTCF has little effect on the recruitment of MRE11 [16]. Therefore, the recruitment of CTCF to DSB sites requires MRE11, but not vice versa. Only the zinc-finger domain fragment of CTCF is rapidly and strongly accumulated to DSBs, like the full-length of CTCF. The N-terminal interacts weakly with MRE11 and thus the N-terminal is very weakly recruited to DSBs. By contrast, the C-terminal fragment of CTCF, which is not able to interact with MRE11, is rarely recruited to DSB sites [16]. These results indicate that CTCF recruitment at DSBs requires its interaction with MRE11 (Figure 4), highlighting the critical importance of CTCF interaction with its binding partners in diverse cellular processes.

CTCF also interacts with CtIP that is a cofactor for the endonuclease activity of MRE11. CTCF depletion dramatically abrogates CtIP recruitment at sites of DNA lesions [16], unlike the MRE11 recruitment that is not influenced by CTCF depletion. Similarly, MRE11-depleteion causes defective CtIP recruitment at DNA lesions. In contrast, knockdown of CtIP had no effect on either MRE11 or CTCF accumulation to DSB sites. Co-immunoprecipitation (Co-IP) experiments demonstrate that zinc finger domain-containing CTCF protein fragments are able to interact with CtIP and can restore the impaired CtIP recruitment and DNA end resection in CTCF-depleted cells [16]. Therefore, CtIP recruitment to DSBs requires CTCF and also its interaction with CTCF (Figure 4), further emphasizing the crucial importance of CTCF interaction with its binding partners in DNA damage response. 

Interactome data sets have enabled the identification of much more interacting partner proteins of CTCF than previously thought (Figure 2 and Table 1), and it may not be a coincidence that CTCF interactions have been implicated in many of its functions identified to date. One example from CTCF interactome data is the tumor suppressor breast cancer type 2 susceptibility protein (BRCA2) that promotes DNA double-strand break repair by HR. Mutations of the BRCA2 gene often increase the risk of breast and ovarian cancers as part of a hereditary cancer. BRCA2 depletion causes hypersensitivity to genotoxic agents and displays defects in HR and, ultimately, the instability of the genome [62,63]. In the early steps of HR, BRCA2 is recruited to DSB regions, followed by recruitment of Rad51 and coating ssDNA with RAD51 to form RAD51 filaments. The ssDNA-RAD1 filaments then search for DNA homology and conduct single strand invasion for homologous pairing to form a D-loop. DNA synthesis occurs within the D-loop, followed by the resolution of the extended structure [64,65].

Upon DNA damaging, CTCF interacts with BRCA2 and then recruits BRCA2 to DSBs [16]. In CTCF-depleted cells, BRCA2 recruitment at DSBs is remarkably diminished. Together, these data propose that BRCA2 is recruited to DSBs in a CTCF-interaction dependent fashion. In response to DNA damage, PARP1 quickly catalyzes poly (ADP-ribosy)lation (PARylation) at DNA lesions and facilitates subsequent recruitment of MRE11 and RAD51 [66]. CTCF is also PARylated by PARP1 and PARylated CTCF engages in the control of gene imprinting and ribosomal gene transcription [67]. Treatment of olaparib, a PARP1 inhibitor, decreases the interaction between CTCF and BRCA2, and the CTCF PARylation-defective mutant is not able to recruit BRCA2 on DSB sites although its DNA binding affinity is not altered (Figure 4) [17]. Therefore, although PARylation of CTCF is not required for DNA binding, it is essential for the recruitment of BRCA2 to DSB sites. 

A large body of evidence strongly supports the functional significance of PARylation in DNA damage response. Regarding its functional interplay with CTCF in addition to BRCA2, recruitment of CTCF is remarkably abolished by olaparib treatment, like BRCA2. These results suggest that PARylation is required for the quick recruitment of CTCF and BRCA2 to DSB sites in an ordered manner. An in vitro PAR binding assay proved that the zinc finger domain of CTCF is sufficient to recognize PAR and accumulate CTCF itself proteins at DNA lesions (Figure 4) [55]. This study demonstrates that CTCF is quickly recruited to DSB sites in a PARylation-dependent fashion and then plays an important role in the early DNA damage response.

Lang et al. also reported that CTCF directly interacts with RAD51 through its C-terminal domain. The formation of RAD51 foci is reduced by CTCF knockdown [18]. This result suggests that CTCF promotes Rad51 repair foci formation by facilitating Rad51 recruitment (Figure 4). Taken together, CTCF functionally interplay with many HR-mediated repair proteins, possibly via their interactions with DDR proteins. 

CTCF depletion increases γ-H2AX foci and induces sensitivity to IR and other DNA damaging agents such as methyl methanesulphonate (MMS), etoposide and camptothecin [16,17,18,55]. Studies using mutants and fragments of CTCF have revealed that the zinc finger domain alone restores all or most defects from CTCF depletion, comparably to the full-length CTCF, and thus most CTCF functions related to DDR are indebted to the zinc finger domain [16,55]. Notably, several lines of evidence link CTCF to error-free DNA repair by HR. CTCF physically interacts with multiple HR repair proteins and is responsible for recruitment of HR proteins at DSBs. Furthermore, CTCF depletion suppresses HR but does not impair canonical NHEJ [16,17,18], revealing that CTCF plays a critical role in DNA repair by HR. 

## 4. Future Questions and Perspectives

A new and exciting role of CTCF in HR has provided us with an opportunity to understand that CTCF is doing more than what has been known and to appreciate its versatility. Yet, the better understanding and the more knowledge of CTCF we have, the more important issues and challenging questions involved in DNA repair and the guarding genome remain to be answered. 

### 4.1. A Genome Repair Tool and Beyond

Evidence to date supports that CTCF is doing more than what has been known. More than two decades after the first identification of CTCF as a DNA binding protein responsible for the regulation of *c-myc* gene expression, the many functions and multiple roles fall into line behind CTCF. Given the ability of CTCF to bind to numerous variants with consensus sequences, it is surprising at first glance that its genome-wide recruitment at sites of DNA lesions can occur throughout the human genome across various DSB induction systems. Additionally, available data are consistent with the notion of a newly assigned role of CTCF in DNA repair via HR and possibly other DNA damage response functions independent of sequence-specific DNA binding mechanisms by simply its interaction and cooperation with other protein partners (see above, such as HR proteins, including the MRN complex, CtIP, BRCA2, and RAD51), which are possibly regulated by covalent modifications, such as PARylation and phosphorylation. Although we propose the “CTCF-interaction-cooperative” mechanism on the basis of these results, it may be too primitive to rule out the possibility for instances where CTCF functions via looping-dependent mechanisms that are linked to its primary role as a genome-wide organizer for the 3D chromatin architecture.

### 4.2. A Guardian of Genome

On the basis of its ability to organize the 3D genome, a conceptually advanced hypothesis is starting to emerge whereby disorganized 3D genome or traditional DNA damages and its consequential 3D genome alternations that are yet to be determined, are timely eliminated. In addition to the elimination of 3D genome alternations, its correct repair/recovery via reorganization has a marked effect on how the DNA sequences under the 3D structure are faithfully inherited to offspring and interpreted for a huge array of cellular processes, hence defining “3D genome integrity” and “a guardian of 3D genome integrity”. In this regard, CTCF could do many more things for the genome, in a manner that relies on its ability to organize the 3D genome and relates to genome integrity. Striking observations have been made in mammalian cells exposed to DNA-damaging insults. In these cells, CTCF was found heavily enriched around the boundaries of γ-H2AX chromatin domains in a nearly identical pattern to TAD boundaries and spreading of γ-H2AX chromatin has been observed outside the demarcated γ-H2AX domains after depletion of CTCF. These observations suggest that CTCF serves as an organizer of the γ-H2AX chromatin domain and maintains a specific chromatin architecture for the DNA damage-induced γ-H2AX domain, more than its role as an architectural border protein of γ-H2AX foci. Indeed, insight into the molecular mechanisms whereby CTCF demarcates the γ-H2AX foci chromatin domain under genotoxic stresses and TAD without DNA damage will be significantly important to understand the 3D genome organization in more detail. Determining whether CTCF alone can delimit γ-H2AX foci chromatin domain in a cohesin-independent manner or alternatively in a manner concerted with cohesin and for what demarcation of γ-H2AX foci will be important toward understanding the detailed as well as broader role of CTCF in DNA damage responses and the underlying mechanisms by which CTCF can reorganize genome structure in response to DNA damage.

Furthermore, knockdown of CTCF results in the formation of γ-H2AX foci without any DNA damage insults, even though γ-H2AX foci are generally formed after DNA damage. In light of the concept of the 3D genome structure in conjunction with these observations, its newly added functional element in faithful maintenance of genome integrity to the classical functions, such as transcriptional activation/repression, enhancer blocking/insulation, V(D)J recombination, and imprinting, may all be relevant to its primary role as a 3D genome organizer.

It could be worth mentioning a peripheral issue that loop-anchor sites occupied by CTCF are a major mutational hotspot in cancer [68,69] and prone to spontaneous (i.e., without exogenous DNA damages) breakage and chromosome rearrangement [70,71,72], might also be linked to its role in 3D genome organization and further intrinsic 3D genome integrity. For examples, a report by Canela et al. discovered that type II topoisomerase (TOP2β)-mediated DSBs occur at boundaries of TAD to relieve looping-induced torsional stress, leading to frequent chromosome translocation in cancer [56,72]. Recently, Gothe et al. propose that 3D genome looping and structure in combination with transcription may inflict harm on genome integrity and contribute to chromosome rearrangement that drives cancer. Although how 3D genome organization influences genome integrity remains poorly understood, these data support the notion that loop anchoring sites required for 3D genome organization could be a source of genome instability [70,71]. In addition, mutations of loop anchoring sites could induce altered 3D genome organization and disrupt 3D genome conformation, which could lead to genome instability-associated diseases including cancer. This model could be coupled to a hypothesis of “3D genome structural instability”.

A Hi-C recent study providing important clues into how the 3D genome organization responds to DNA damage suggests that radiation-generated DNA damage induces TAD boundary strengthening, which means an increased segregation of TADs, in an ATM-dependent manner [73], which is consistent with the ATM-dependent CTCF recruitment at DSBs [18] for defining TAD borders in response to DNA damage [29,52]. The DNA damage-induced TAD border strengthening is in agreement with the confinement of the γ-H2AX domain within a TAD in which DSBs take places (see the section entitled “*Colocalization of CTCF with γ-H2AX chromatin to delimit γ-H2AX chromatin domain”*) and propose a hypothesis that 3D genome structure integrity during repair depends on ATM. Another super-resolution imaging study using 3D structured illumination microscopy (3D-SIM) with labelled probes for TAD sequences, allows visualization of 3D chromatin architecture at DNA ends occupied by 53BP1 (p53-binding protein 1), thereby organizing a functional module to stabilize 3D genome topology at broken DNA ends [74], providing a detailed snapshot on the broken DNA ends in the native 3D genomic context. Taken together, these data in both of the Hi-C and 3D-imaging studies support the “3D genome arrangement and organization in response to DNA damage”, and may have been broadened to account for the “3D genome integrity”. 

Accordingly, we could envision the “genome organizer for 3D genome integrity” mechanism whereby, since CTCF depletion indeed impairs 3D genome architecture [54], such a loss of 3D genome structural integrity in turn would be recognized as a kind of DNA damage stress, and, consequently, γ-H2AX foci are formed [18]. Recent reports have also made it clear that CTCF is required for chromosomal integrity. Indeed, depletion of CTCF induces chromosomal breakage and chromosome end fusions. Consistently, remarkable increases in nuclear buds, nucleoplasmic bridges, micronuclei and apoptosis, which are evidence of chromosomal instability, are readily observed in CTCF-depleted cells. Moreover, DNA lesions and chromosomal aberration are highly increased by exogenous DNA damaging stimuli such as exposure to IR, UV and etoposide, in CTCF-depleted cells, which results in apoptosis through the progression of mitosis disregarding the damage-induced G2/M arrest [17,18]. Accordingly, there is strong evidence that CTCF ensures chromosome integrity, further extending 3D genome integrity. Although its exact mechanisms still need to be deciphered, a tempting hypothesis could be a good explanation for a new role of CTCF in 3D genome integrity.

### 4.3. Other Jobs in DNA Damage Responses?

Whether CTCF may continue working, stop its work until recovery, or work hard across its many jobs (such as genome organization via looping, transcriptional repression/activation, insulation, and imprinting), in response to endogenous and environmental DNA damaging cues, remains to be determined. During a vast array of cellular processes in DNA damage responses, CTCF may be involved in transcriptional regulation and cell-cycle checkpoint/arrest via genome reorganization, in addition to DNA repair, whereby cells determine its fate, such as survival/death/disease/senescence, which can account for the higher sensitivity of the CTCF-depleted cells than that of the CtIP-depleted cells to etoposide [16].

### 4.4. Candidate Collaborators of CTCF in HR

Numerous protein interactors of CTCF, including many essential HR factors, have been described [16,17,18]. It is surprising that BRCA1 is not in this list. Furthermore, CTCF does not function alone; it has to cooperate with various HR factors, which elegantly coordinate the complex repair processes. Furthermore, BRCA1 is absolutely required for multiple stages throughout HR pathway processes, particularly in light of the most essential leverage and crucial importance of BRCA1 for HR pathway. Although they do not interact directly, it is apparent that they could, at least indirectly, interact physically with each other and may collaborate in HR, because depletion of either one severely impairs HR. Yet, where and how they functionally interplay to successfully conduct HR remain elusive. Otherwise, how do they relate to the different HR steps? Most steps of all the repair reactions and processes throughout are aided by HR. Particularly before all the later stages of HR, whether and how CTCF and BRCA1 collaboratively function in the initial step (that is the most crucial choice step) of the HR pathway and the generation of ssDNA tail via end resection, which is aided by both CTCF and BRCA1, remain to be determined. 

Conversely, CTCF interacts physically and directly with RAD21 (also known as SCC1), that is a subunit of the cohesin complex and regulates DNA repair as well as chromosome segregation [75,76]. Several studies revealed that RAD21 and SCC3 (also known as SA2 or STAG2) are required for HR in human cell lines [75,77], previously characterized as recruited at DNA lesions [57,78,79,80,81]. Hi-C study has shown that cohesin is responsible for a search for DNA homology and sister chromatid exchanges in yeast cells [82]. However, the underlying mechanisms and accurate functions of cohesin subunits of HR in mammalian cells are still up debate and remain unclear. In vitro as well as in vivo functional and biochemical investigations are required to better understand whether and how CTCF collaborates with Rad21 and/or SCC3 in HR.

### 4.5. Concluding Remarks

Recent methodological developments relevant to and combined with chromosome conformation capture, advanced proteomics, and live super-resolution microscopy have enabled elucidation in great detail of how CTCF organizes the 3D genome via concerted actions with other architectural proteins and DSB repair proteins, including HR factors. This information will undoubtedly increase our understanding of the spatiotemporal organization of the genome with or without DNA damage. In this regard, the integration of 3D genome architecture and DSB repair fields will innovatively provide investigators in the two fields with significant insights into genome organization and the underlying mechanisms whereby CTCF coordinates 3D genome organization and ensures genome integrity. 

## Figures and Tables

**Table 1:** The protein partners of CTCF.

Protein Partner		Functions of CTCF–Protein Interaction	Refs
Transcription enzyme	RNA polymerase II large subunit (LS pol II)	Regulation of transcription and insulator functions	[23]
Transcription regulatory factor	CIITA	HLA-DRB1 and HLA-DQA1 gene transcription	[23,24]
	Regulatory factor X (RFX)	HLA-DRB1 and HLA-DQA1 gene transcription	[23,24]
	Kaiso	Regulation of insulator functions Transcriptional repression of RB gene following CTCF-binding sites methylation	[23,24]
	Oct4	X chromosome pairing and counting	[23]
	CTCF	Interaction between distant DNA regions	[23]
	YB1	Transcriptional repression of c-myc and serotonin transporter (5-HTT) gene	[23,24]
	YY1	Tsix gene transactivation	[23,24]
	BPTF	Transcriptional suppression of H2-K1 gene via inhibition of Klf4 binding	[25]
**Chromatin constituent**	CHD8	Regulation of insulator functions	[23,24]
	Suz12	Regulation of insulator function (H19 ICR)Transcriptional repression of Sox2	[23,24]
	Sin3A	Transcriptional repression via recruitment of HDAC	[23,24]
	Taf-1/Set	Unknown	[23,24]
	H2A/H2A.Z	Co-localize genome-wide	[23,24]
	HP1α	Interact in pericentric heterochromatin (PCH) and restricts H4K20me3 and H3K27me3 distribution	[26]
**Genome integrity**	PARP1	Regulation of crosstalk between poly(ADP-ribosyl)ation and DNA methylation	[23,24]
**RNA binding protein**	Vigilin	Interacts with CTCF via H19 lncRNA and keep the imprinting of IGF2	[27]
**Nuclear architecture**	Nucelophosmin	Regulation of insulator function	[23]
	Cohesin	Co-localize genome-wide Regulation of insulator function (c-myc, H19/Igf2)	[23,24,29,30]
	Lamin A/C	Unknown	[23]
	Topoisomerase II	Unknown	[23,28]
**DNA damage repair**	MRE11	CTCF and CtIP recruitment on DNA double strand break (DSB) sites	[16]
	CtIP	Induction of DNA end resection	[16]
	RAD51	Homologous pairing and strand invasion	[18]
	BRCA2	Assembly of RAD51 on single stranded DNA	[17]
	PARP1	Chromatin PARylation and early recruitment of CTCF to DSB sites	[17]

## Abbreviations

CTCF	CCCTC-binding factor
DSBs	double-strand breaks
NHEJ	non-homologous end joining
HR	homologous recombination
IR	ionizing radiation
UV	ultraviolet
DDR	DNA damage response
MRN	MRE11-RAD50-NBS1
ssDNA	single-stranded DNA
D-loop	displacement loop
ChIP	chromatin immunoprecipitation
ICR	imprinting control region
TCR	T cell receptor
CBE	CTCF-binding element
TAD	topologically associated domain
PAR	poly(ADP-ribose)
